# Beyond confined catalysis in porous materials

**DOI:** 10.1093/nsr/nwaa044

**Published:** 2020-03-14

**Authors:** Xiaoliang Wang, Pui Ching Lan, Sai Wang, Shengqian Ma

**Affiliations:** Department of Chemistry, University of South Florida, USA; Department of Chemistry, University of South Florida, USA; Department of Chemistry, University of South Florida, USA; Department of Chemistry, University of South Florida, USA

The assembly of active species into porous material has been extensively investigated in the past decades [1]. Especially with the utilization of Metal-Organic Frameworks (MOFs), the techniques of encapsulating active species have been widely explored, which is beneficial to the unique aspects of MOFs, like tunable porosity, high capacity, open active sites and crystallinity nature, etc. [2]. With the case for trapping soluble active molecules, it is commonly believed that homogeneous catalysts have better catalytic performance than heterogeneous catalysts because of higher dispersion of active sites [3]. Given this, the assembly of host porous material and active molecule guest becomes crucial. This integration is supposed to meet several key requirements: (i) the assembled system must contain a large interior cavity for the homogeneous catalyst; (ii) the porous host should have the size-selectivity to allow substrates/products transport and restrain the active guests from leaching out; (iii) the valuable and practical properties of host material and guest molecules should be highly retained.

In a recent work published in *National Science Review*, Prof. Hai-long Jiang at the University of Science and Technology of China and co-workers created a yolk-shell MOF capsule (noted YSMCs) for integrating the heterogenous host and homogenous catalyst in one system [4]. In this study, the hollow template, named layered double hydroxides (LDHs), provides a large opening and hollow cavity for homogeneous catalysts and then the MOF shells with well-defined micropores were fully covered onto the hollow template of the LDH after entrapping the active species inside (Fig. 1). Typically, works that employed MOFs or other porous materials as platforms are utilizing the micro or mesopores of MOFs to directly incorporate active species and the total pore volume is greatly decreased due to active guests obstructing pores [5]. Meanwhile, the leaching of encapsulated guests, in most cases, could be detected because of inefficient confinement or weak interaction with the interior surface. Those deficiencies will eventually impact the performance of catalytic activity and dwindle recyclability [5].

**Figure 1.**
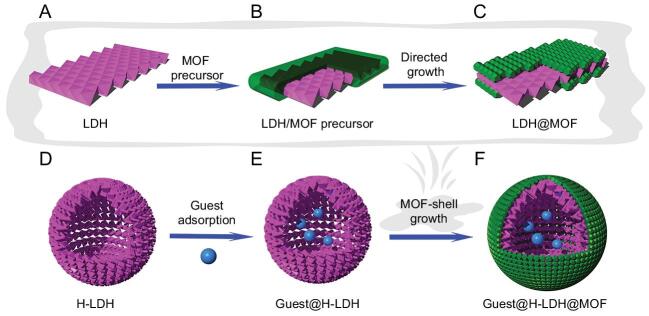
Schematic for the fabrication of the yolk-shell MOF capsule. (A-C) The template-assisted strategy to create LDH@MOF and (D-F) encasing guest molecules (blue sphere) into H-LDH by creating a yolk-shell MOF capsule. Redrawn from Fig. 2 of [4].

The directed strategy of growing MOFs on the template LDH is taking advantage of the positively charged scaffolds of unsaturated metal sites and negatively charged deprotonated ligand to fabricate a specific shell thickness. In order to demonstrate the versatility of this strategy, various MOFs like ZIF-8, ZIF-67 and MOF-74 were

attempted and successfully assembled on the surface of LDH templates with continuous coverage. To investigate the molecular-size-selectively permeable function toward encapsulated active species, differently sized molecules, such as Coomassie Brilliant Blue R250 (R250) and n-octylamine, were employed and further studied about the uptakes and release of incorporated active species from H-LDH@ZIF-8. It experimentally demonstrated that there is no restricted mass transport of the MOF shell for small molecules, n-octylamine. In addition, the authors embedded various homogenous catalysts in H-LDH@ZIF-8 to form YSMCs, in which a perfect MOF shell not only retains the high intrinsic activity of the molecular catalysts and suppresses their leaching, but also endows the resultant composites with substrate enrichment, size-selectivity and multifunctional cascade catalysis.

The presented work remarkably addressed the issues of compromised property by creating a specialized nano-capsule reactor with soluble yolks and perfectly crystalline porous shells. This allows the entrapped homogeneous catalysis to entirely proceed in the confined space and imposes high porosity for size-selective mass transport. It opens a new pathway to elegantly integrate heterogenous and homogenous in one system and retain the properties of the host template with a MOF shell and active species. The template-assisted strategy like the yolk-shell MOF capsule will significantly extend the construction and application of catalysts in porous materials.

***Conflict of interest statement***. None declared.

## References

[1] Leenders SH , Gramage-Doria R, De Bruin B et al. Chem Soc Rev 2015; 44: 433–48. 25340992 10.1039/c4cs00192c

[2] Horcajada P , Gref R, Baati T et al. Chem Rev 2012; 112: 1232–68. 22168547 10.1021/cr200256v

[3] Ren J , Lan PC, Chen M et al. Organometallics 2019; 38: 3460–5.

[4] Cai G , Ding M, Wu Q et al. Natl Sci Rev 2020; 7: 37–45. 10.1093/nsr/nwz147 PMC8288971 34692015

[5] Li G , Zhao S, Zhang Y et al. Adv Mater 2018; 30: 1800702. 10.1002/adma.201800702 30247789

